# Why the preclinical imaging field needs nuclear medicine technologists and radiographers?

**DOI:** 10.1186/s41824-020-00081-z

**Published:** 2020-07-20

**Authors:** J.-P. Dillenseger, P. Choquet, E. R. Snay, P. Fragoso Costa

**Affiliations:** 1 ICube (MMB, AVR)-UMR 7357, CNRS, Université de Strasbourg, Strasbourg, France; 2 Imagerie Préclinique-UF6237, Hautepierre, Pôle d’imagerie, Hôpitaux Universitaires de Strasbourg, Strasbourg, France; 3 Fédération de Médecine Translationnelle de Strasbourg, Faculté de Médecine, Université de Strasbourg, Strasbourg, France; 4 Laboratory for Adaptable MRI Technology, Department of Biomedical Engineering, University of Basel, Basel, Switzerland; 5 Department of Radiology, Boston Children’s Hospital and Harvard Medical School, Boston, MA USA; 6 Department of Nuclear Medicine, University Hospital Essen, University of Duisburg-Essen, Essen, Germany

## Abstract

**Introduction:**

Preclinical imaging is still seen as a new field, and its recognition as a specific topic occurring only about 20 years ago. Nuclear medicine technologists (NMTs) and radiographers’ skills covering technical, anatomical and clinical fields can be highly beneficial to preclinical imaging research centres: many tasks and knowledge are complementary between clinics and preclinical laboratories. Our goal is to reach a consensus on the required set of competencies needed to translate the work of NMTs and radiographers from the clinic to the preclinical laboratory, particularly in regard to multimodal imaging.

**Preclinical imaging environment:**

Currently, all imaging modalities used in clinical routine (ultrasound, CT, MRI, PET, SPECT, radiographs) are available, using specific architectures allowing for the spatial resolution and sensitivity needed for small rodents (which are the most commonly used species in research). Ideally, a preclinical laboratory should produce images/examinations at a high throughput in order to meet the statistical expectations of the studies (while respecting the 3R principles for animal research) and the care and welfare of each individual. To reach the quality and throughput expectations of such an organization, specific qualified professionals are needed to complete the scientific/research staff.

**Where NMTs and radiographers fit in:**

The increasing use of preclinical imaging requires professionals who can put imaging procedures into action, ensuring a significant success throughput. NMTs and radiographers have a variety of skills that work well within a preclinical laboratory, with the ability to perform the following tasks independently: animal preparation, positioning, monitoring and anaesthesia recovery, acquisition parameter programming, archiving and data processing, device quality controls, surface cleaning and disinfection, radioactive and biological waste management, radiation safety for users, use of hot lab equipment and auxiliary equipment, injected products and material management. In light of the current European Qualification Framework, a set of skills, knowledge and competencies were defined to cover the whole set of duties and tasks deliverable to an NMT or radiographer working in a preclinical laboratory. One of the key responsibilities of the NMT or radiographer is related to compliance on animal care and welfare when undertaking any animal procedures, including imaging.

**Conclusion:**

We believe that NMTs and radiographers’ skills match perfectly with the requirements of a preclinical imaging lab, and that they could be considered a keystone of such an organization in the future. Moreover, some evidence has also shown that an experienced NMT or radiographer in this sector can take on roles as research investigators.

## Introduction

Preclinical imaging is still seen as a new field, and its recognition as a specific topic occurring only about 20 years ago. Although MRI was developed and used for imaging lab animals as early as the 1980s, the concomitant technical achievement of adapted instruments for small animal imaging for all major imaging modalities (x-ray tomography, positron emission tomography (PET), single photon emission computed tomography (SPECT), ultrasound) enables the birth of a new imaging field with specific requirements. Clinical nuclear medicine technologists (NMTs) are recognized by the European Association of Nuclear Medicine (EANM) and the International Atomic Energy Agency (IAEA) when referring to health care professionals who have contact with patients (as opposed to technicians) and are able to undertake the whole range of nuclear medicine diagnostic and therapeutic procedures under the direction of a nuclear medicine physician (Fragoso Costa et al. 2017). Clinical radiographers are defined by the European Federation of Radiographer Societies as medical imaging and radiotherapy experts who are accountable for the patients’ welfare during, prior and following examinations, while taking active roles in the justification and optimization principles of radiation protection. In this paper, we refer to NMT and radiographers interchangeably, as clinical professionals who have no curricular animal handling training, contrasting to animal or lab technicians. With the exception of preclinical laboratories built within university hospitals, the involvement of NMTs and radiographers remains limited, as their skill sets are widely unknown outside of academia. Indeed, NMTs and radiographers’ skills covering technical, anatomical and clinical fields can be highly beneficial to preclinical imaging research centres: many tasks and knowledge are complementary between clinics and preclinical domains. These aspects were presented and debated during the European Association of Nuclear Medicine (EANM) congress in 2014 (Dillenseger et al. 2014) and recently at the European Congress of Radiology 2018 (ECR18) in a special-focus session, “Technologists and radiographers in Preclinical Research” (Dillenseger 2018), which proves that this subject is topical and of interest to a wide range of professionals (researchers, radiologists, etc). During these sessions, it was acknowledged that technologists and radiographers, given their technical, practical and methodological expertise, would be great assets to preclinical teams. In this integration, NMTs and radiographers could have an entrance into research and academic fields.

The goal of this paper is to introduce NMTs and radiographers to the underlying principles of preclinical research, particularly in regard to imaging, and to emphasize their potential role in this field. Furthermore, our intention is to reach a consensus on the required set of competencies needed to translate the work of an NMT or radiographer from the clinic to the preclinical laboratory, focusing in multimodality imaging.

## What is preclinical research?

**Fig. 1:**
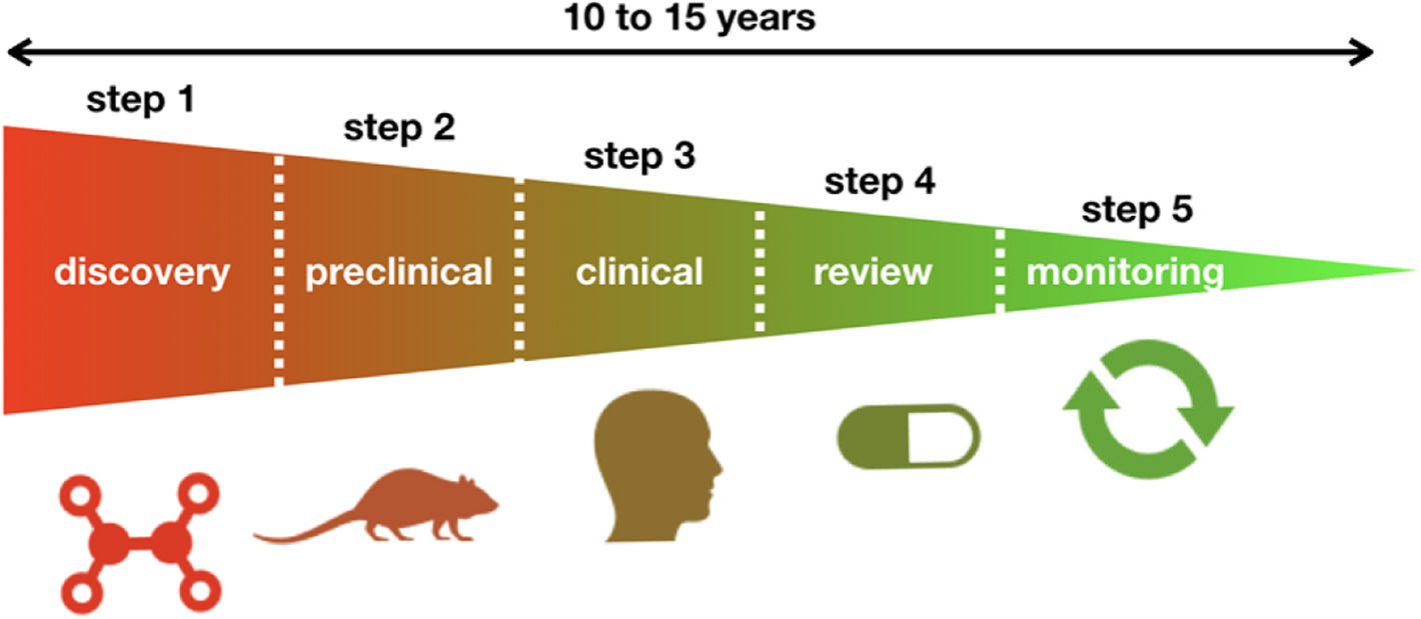
The 5 steps from the drug development process

Preclinical research concerns all studies carried out on laboratory animals to observe and understand biological phenomena but also to test new exploration procedures. It is most often included and defined as a step (preceding the clinical phases) of the drug development process (Fig. 1):

- Step 1. Discovery and development (fundamental research)
- Step 2. Preclinical research (tests on animals)
- Step 3. Clinical research (tests on humans) which involves 4 sub-steps (phase I to phase IV)
- Step 4. Review from regulatory agencies (e.g. FDA)
- Step 5. Post safety monitoring from regulatory agencies

Preclinical research (step 2) addresses basic questions about safety and efficacy of novel drugs, therapeutic procedures and treatments using animal models (Begley and Ellis 2012). This preclinical step necessarily precedes the transition from a “therapeutic technology” to phases of clinical testing in humans. In 2002, sequencing techniques demonstrated that the genomes of mice and humans are very close (Chinwalla et al. 2002). This discovery, coupled with the achievement of specific small-animal imaging systems developing in the early 90’s (e.g. μPET especially) (Kiessling and Pichler 2016), placed the mouse model as a standard in animal experimentation. Since then, small-animal preclinical research has increased with the objective of facilitating the delicate and complex transition from mice (preclinical research) to humans (clinical research) referred to as translational research (Woolf 2008). Although preclinical research deals especially with the development and study of new drugs, preclinical imaging became the usual way to talk about small laboratory animal imaging regardless of the application. Fundamental research tends to use these methods as well, and due to the advantage of longitudinal studies, the ability to follow the same individual in vivo along their lives or pathological course. Preclinical imaging names all applications.

Although many different species are employed, the use of mouse animal models has several practical and scientific advantages:

- Short life cycle
- Simple and space-saving rearing
- High prolificity (average gestation duration 19–21 days)
- Genetic similarity to humans

In 2017, in the EU, 92% of the animals used in research and testing were mice (61%), fish (13%), rats (12%) and birds (6%), “while species of particular public concern (dogs, cats and non-human primates) represented less than 0.3% of the total number of animals” (https://ec.europa.eu/environment/chemicals/lab_animals/reports_en.htm). The uses of these animals are allocated across fundamental research (45%), translational and applied research (23%) and regulatory use (23%).

Genetic modification techniques (transgenesis) are now well mastered in mice, which offer a wide range of pathological models. In fact, some establishments are now devoted exclusively to the breeding of mice and the development of pathological models (Lyons 2005).

Of course, the use of a large number of animals (9.58 million in 2017 in the EU) raises ethical questions. Thus, recommendations exist and should be rigorously applied as the ethical rule of the 3Rs (Russell et al. 1959) which imposes on researchers to:

- Reduce as much as possible the number of animals used for a given experiment
- Refine experimental methodologies to obtain maximum information from each experiment and be minimally invasive as possible
- Replace the use of animal models when alternatives are possible (cell cultures, bioinformatics simulation, *inter alia*).

Translated in regulation, application of these rules allows a reduction in the number of animals while maintaining the validity of experiments. Consequently, reduction and refinement rules promote the use of non-invasive techniques such as imaging. Thus, preclinical imaging is expanding. Due to morphological and physiological differences (not only based on the size) between mouse and human (Table 1), preclinical imaging requires the use of dedicated systems. This is not the case with larger species (rabbits, pigs, dogs or non-human primates) all of which fit inside clinical instruments, once equipped with ancillary equipment required for specific physiological set up (e.g. dedicated anaesthesia system).

**Table 1:** Average anatomical and physiological data of adult mice compared to humans.

	***Mus musculus***	***Homo sapiens***
**Average weight**	18–25 g (female) 20–40 g (male)	62.4 kg (female) 77.4 kg (male)
**Average length**	7 à 11 cm	1 m 63 cm (female) 1 m 76 cm (male)
**Number of newborn (pregnancy)**	6–8 (average)	1 (96.54%) 2 (3.31% “twins”) > 2 (0.15%) (US in 2010)
**Average gestation duration**	19–21 days	268 days
**Internal temperature**	37.0–37.2 °C	36.5–37.5 °C
**Average heart rate (beat per minute: bpm)**	310–840 bpm mean 600 bpm	60–70 bpm
**Average respiratory frequency (cycle per minute: cpm)**	84–230 cpm mean 160 cpm	12–20 cpm
**Specific blood volume**	79 ml/kg	77 ml/kg
**Average blood volume**	2 ml (for 25 g)	5.4 l (for 70 kg)
**Glomerular filtration rate**	24 ml/min 12 blood volume/min	100 ml/min 0.02 blood volume/min
**Diameter main bronchus**	1 mm	10–15 mm
**Ventilation**	23–47.5 ml/min	6000 ml/min
**Total body water (TBW)**	72.7% (male) 68.5% (female)	60% (male) 50% (female)

The glomerular filtration rate of the mouse makes it possible to filter the blood mean volume of the animal in approximately 5 s (whereas ≈ 60 min for human beings).

Recently, zebrafish has emerged as a promising in vivo model by offering opportunities to quickly screen recent drug developments under in vivo conditions and in a cost-effective manner so as to bridge the current gap between in vitro and rodent studies (Sieber et al. 2019). Zebrafish has also become a powerful vertebrate model for genetic studies of embryonic development, organogenesis and increasingly for studies in cancer biology (Konantz et al. 2012). Hence, why more zebrafish models are being used in preclinical imaging (De Jong et al. 2014).

## What is preclinical imaging?

**Fig. 2:**
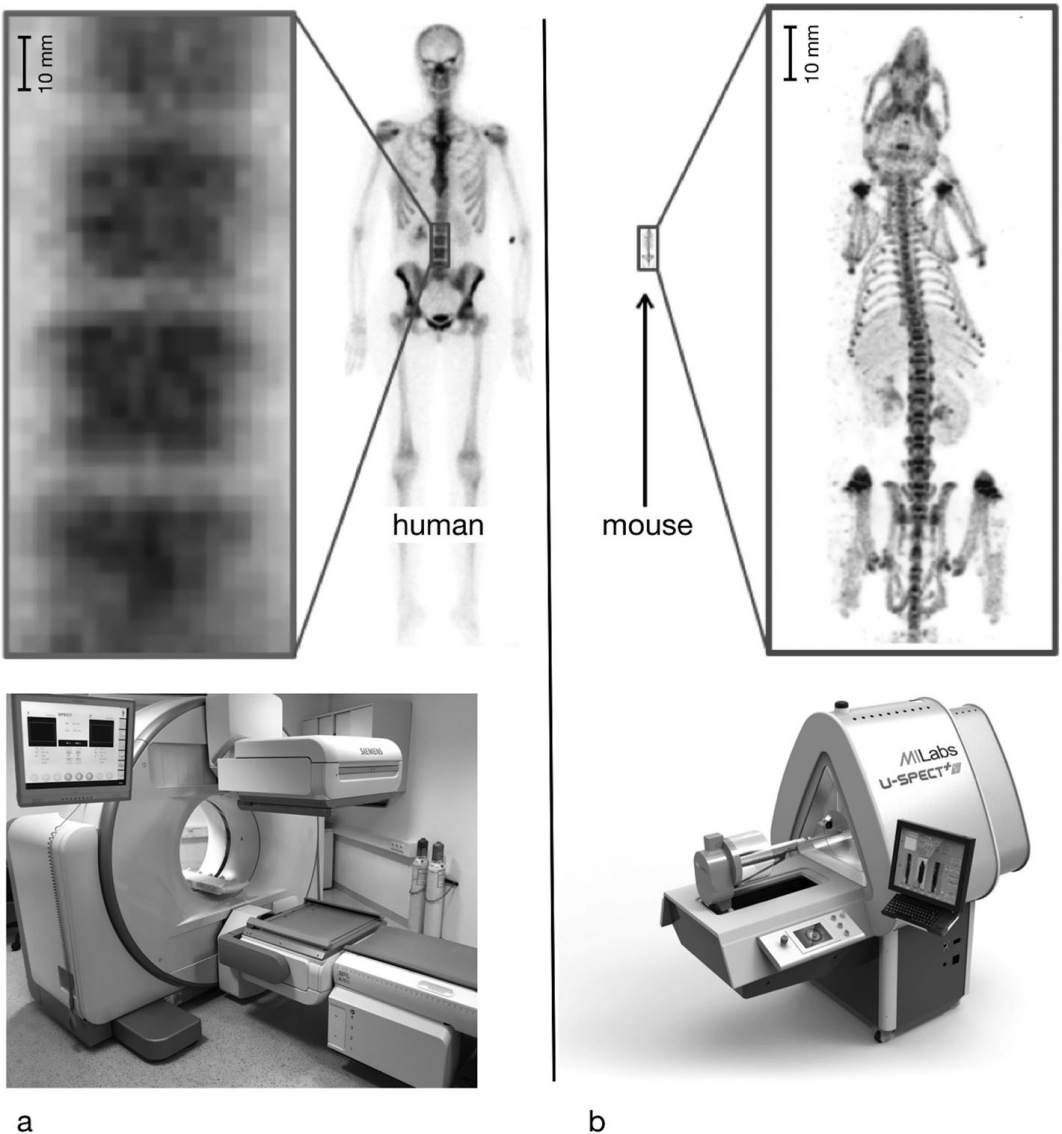
Spatial resolution performance comparison in SPECT between a clinical (**a**) system and a preclinical system (courtesy of MILabs B.V) (Ivashchenko et al. 2015) (**b**)

Currently, all imaging modalities used in clinical routine (ultrasound, CT, MRI, PET, SPECT, radiographs) are available, using specific architectures allowing for the spatial resolution and sensitivity needed for small rodents (Fig. 2). The animals’ size also allows access to other whole body modalities which are difficult to use with humans in the same manner, such as optical imaging (bioluminescence, fluorescence) and photoacoustic imaging (Kiessling and Pichler 2016).

The implementation of preclinical imaging over the past 20 years has allowed morphological and functional explorations at different scales (molecules, cells, tissues, organs, systems, whole body) which explains its expanding use in research.

**Fig. 3:**
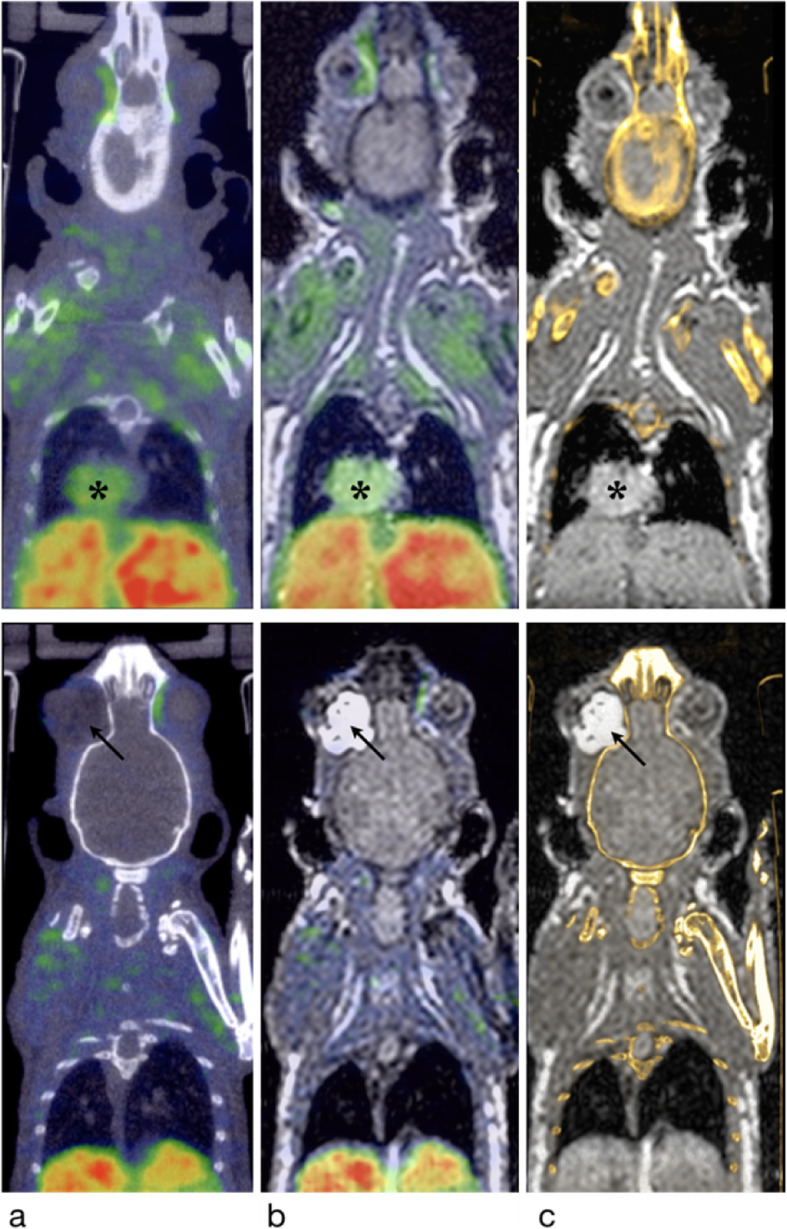
Coronal slices from a multimodal protocol: 1.5 T MRI (T1w)-μCT-μSPECT (^99m^Tc-MIBI) on a mouse (C57BL6) with spontaneous mediastinal tumours (black star) and retro-orbital tumour (black arrow). μSPECT/CT (**a**), SPECT/MRI (**b**), and μCT/MRI (**c**) coregistrations

**Fig. 4:**
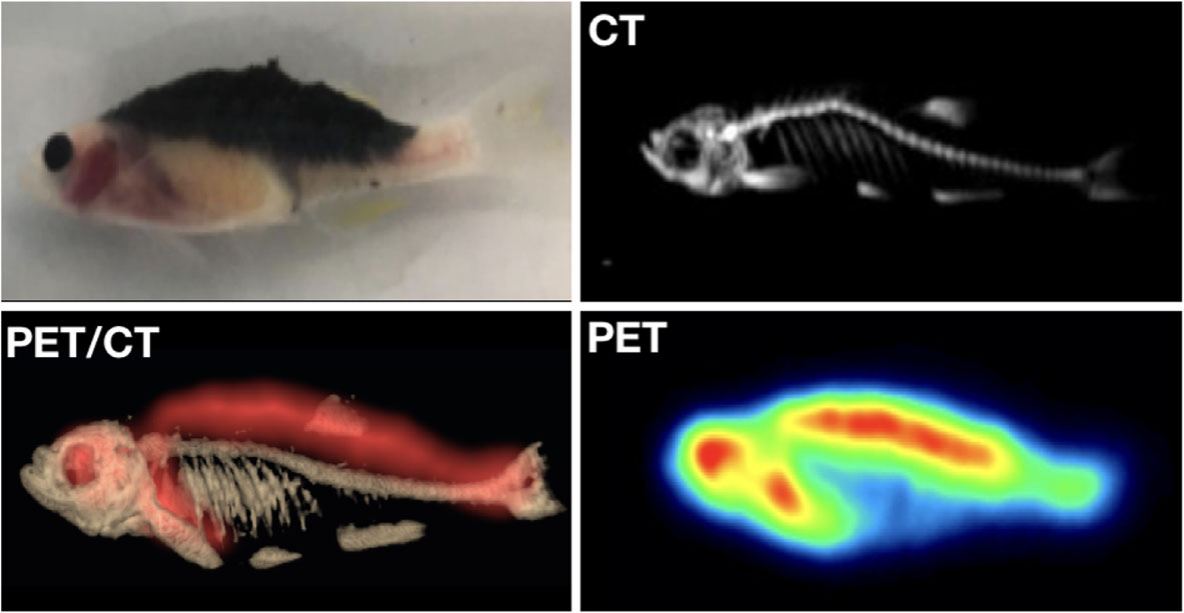
FDG PET/CT of zebrafish with a dorsal tumour imaged at 28 days

This growth has naturally led to the emergence of many technical labs dedicated to small animal imaging, in the pharmaceutical industry, but also in public research laboratories. In the latter case, they are often based in universities, with few preclinical labs even being directly located in university hospitals (Dillenseger et al. 2013). These labs use several imaging modalities daily. In general, they are equipped with anatomical modalities (e.g. μCT and MRI) and functional modalities (e.g. μSPECT, μPET and optical imaging) via separate or combined devices (e.g. SPECT-CT, PET-CT and PET-MRI). Multimodality is essential in current clinical and preclinical imaging. Figure 3 illustrates the possibilities/diversities of multimodal approaches (μCT, μSPECT, MRI) in mice by combining, in this example, spontaneous mediastinal and retro-orbital tumour acquisitions, and Fig. 4 illustrates a zebrafish explored in μPET/CT.

Unlike clinical imaging, preclinical imaging is not primarily intended to provide a diagnosis. For instance, the pathological process (e.g. cancer cells) is generally induced voluntarily, and the objective of preclinical exploration remains mainly the longitudinal follow-up of a group of individuals, or even an associated control group. Physiological processes are also explored in normal or transgenic individuals. In this context, preclinical imaging demands following a large number of individuals (respecting the 3R rules) over time. This method requires considering preclinical imaging devices as reliable measurement tools with necessary regular quality controls. Ideally, a preclinical laboratory should produce images/examinations at a high throughput in order to meet the statistical expectations of the studies, but also for economic reasons while respecting the 3R rules and maintaining appropriate and ethical management of each animal individually. This way of working is the same as in a clinical imaging department, where workflow should be maintained as well as patient care. To reach the quality and throughput expectations of such an organization, specific and qualified professionals are needed to complete the scientific/research staff, and NMTs and radiographers are ideally suited.

## Where NMTs and radiographers fit in?

Early on, preclinical imaging involved mainly physicists, chemists and engineers. Their scientific and technical skills are now progressively complemented by others coming from the biological and clinical fields. Thus, increasingly, more clinicians have developed preclinical laboratories to establish links between clinical and experimental approaches. The increasing use of preclinical imaging requires professionals who can put imaging procedures into action, ensuring a significant success-throughput required to obtain high statistical power, while taking care of each individual as a patient. NMTs and radiographers have the experience and skills to occupy these positions. In the process of translating from clinics to a preclinical environment, the NMTs and radiographers must acquire specific legal and training developmental competencies on the handling and care of laboratory animals. Such training must be adapted to the target animal experimentation species, as welfare needs vary for different species. It is fundamental that a close bond is built between the designated veterinarian, animal welfare officer and staff, as to successfully transfer the fundamental knowledge and responsibility in respect to animal welfare in the preclinical setting.

Although NMTs and radiographers are trained in the use of clinical imaging equipment, an adaptation period is necessary for learning the use of preclinical devices designed specifically for small animals, with software interfaces being less user-friendly in general than those encountered in the clinical field.

Even if NMTs and radiographers have a significant number of skills (England et al. 2017) that can be transferred directly to preclinical imaging, additional training is mandatory concerning the handling and care of laboratory animals for regulatory, ethical and scientific reasons. A multidisciplinary strategy in close cooperation with veterinaries and animal staff must be encouraged in order to increase logistics effectivity and expand the NMT and radiographer responsibilities.

Each individual should be considered as a patient and treated as such. Maintaining homeostasis under experimental conditions could be challenging, as even small differences, for instance, in temperature, could lead to dramatic quantitative discrepancies (Goetz et al. 2017). Applying precise procedures and taking care of each individual is fundamental for obtaining reliable data and following good research practice (Bespalov et al. 2019).

In fact, there are specific regulatory requirements for the training and development of competence for anyone undertaking any work with laboratory animals. Complying with transparent scientific reporting (Kilkenny et al. 2010) and ensuring that all studies are planned and executed with appropriate experimental designs are requirements (Smith et al. 2018).

In the EU, any work must be in full compliance with the EU Directive 2010/63/EU and also supported and approved by the institutional animal ethical body or the Institutional Animal Care and Use Committees (IACUCs) in the USA. So, based on this robust legislative framework, the radiographer or NMT involved in a preclinical imaging lab holds a real professional responsibility to ensure compliance and should also be involved in education in this field.

**Fig. 5:**
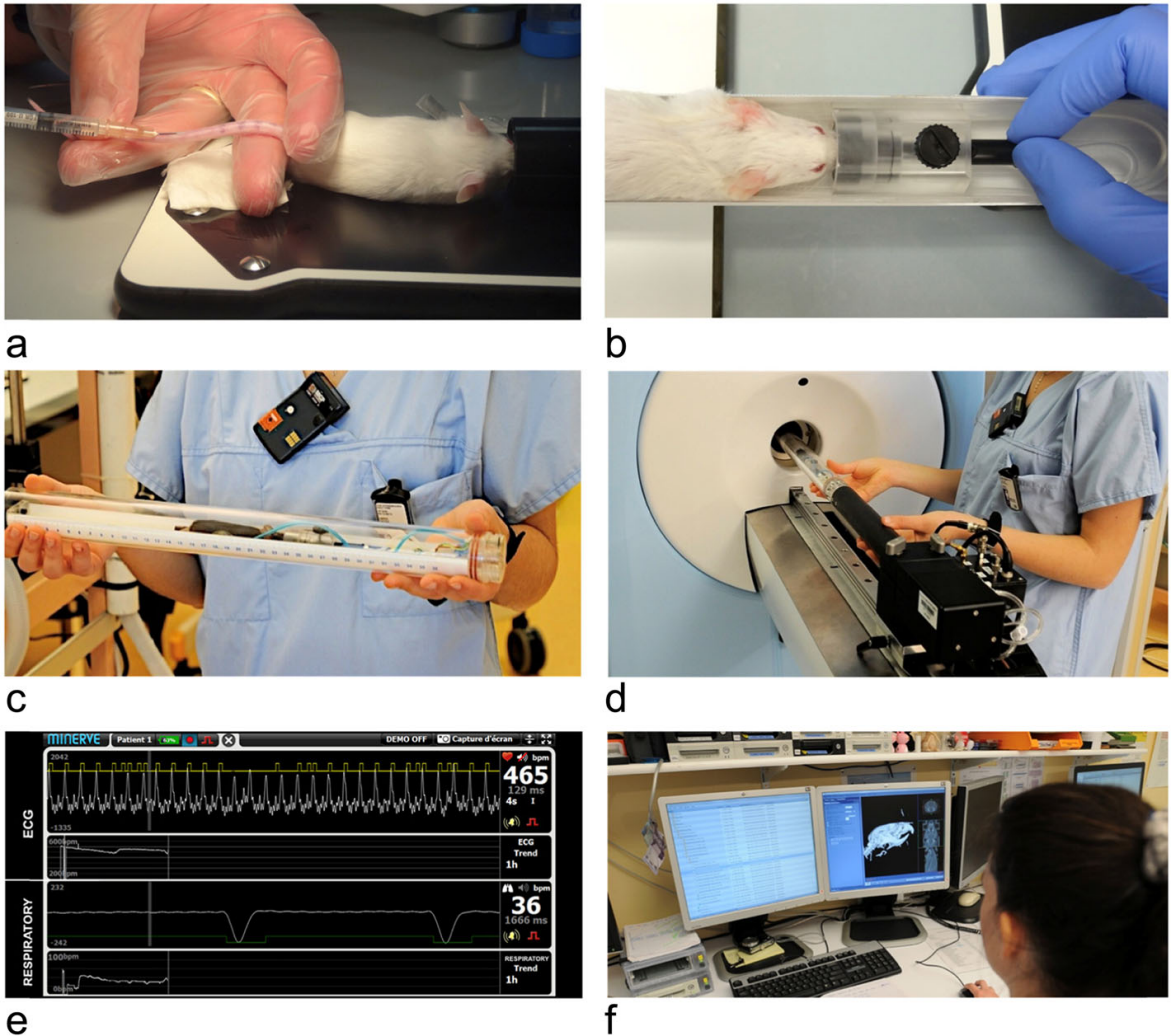
Examples of procedures performed by NMTs and radiographers in a preclinical department. Intravenous injection (**a**), animal anaesthesia and positioning (**b**) in an imaging cell (**c**). Imaging cell docking (**d**), animal monitoring (**e**), data acquisition, archiving, and post-processing (**f**)

As a consequence, the holding of a training certificate in animal experimentation is necessary to meet the regulatory restriction, though requirements vary regionally and nationally. Once these restrictions have been overcome, NMTs and radiographers can advance to perform the following acts independently (Fig. 5):

- Animal preparation (anaesthesia, installation of vascular approaches, monitoring)
- Animal positioning in imaging cell and in imaging devices
- Animal monitoring during a procedure
- Acquisition parameters, programming and setting
- Archiving and data processing
- Anaesthesia recovery
- Post-awakening animal management
- Device quality controls
- Surface cleaning and disinfection
- Radioactive and biological waste management
- Radiation safety (for users)
- Use of hot lab equipment and auxiliary equipment
- Injected products and material management

In some instances, the duration of preclinical imaging procedures could present some additional challenges when compared to clinical scanning. For instance, a complete preclinical procedure (from the preparation of the animal to the end of the data acquisition) including 2 imaging modalities (ex: PET and MRI) can reach > 90 min. If multiple animals are being imaged, these can be even longer and challenging to schedule appropriately. One solution to this has been the use of 3D printed beds, referred to as hotel imaging. This allows the researcher to image 2–4 animals at a time (Greenwood et al. 2019; Dazai et al. 2011).

Thanks to their technical and clinical experience, NMTs and radiographers can easily position themselves in this kind of structure. In fact, most laboratories that have integrated NMTs and radiographers confirm that their technical expertise and practical knowledge improves the flow of acquisitions, which allows researchers to be more focused on more academic aspects (bibliographic research, analysis of results, communication and scientific articles preparation); thus, the NMT and radiographer can be considered a “research facilitator” (Fritsch and Dillenseger 2015).

The preclinical laboratory setting can benefit from the practical expertise of NMTs and radiographers for the development of procedures that are as close as possible to clinical practices. This facilitates the translation of results from animals to humans and could even check the feasibility and the soundness of a suggested protocol. Moreover, a radiographer or NMT could promote specific research on adequate imaging procedures and be able to respond to fundamental or practical scientific questions. This is why NMTs and radiographers could also be involved in staff meetings as “practical experts” and as research “collaborators” (Harris and Paterson 2016). Indeed, the translational and multidisciplinary aspect of preclinical research, should result in proactive clinic-based initiatives, that in its turn result in optimised procedures for the benefit of the animals subject to imaging.

To date, despite their considerable strengths, the involvement of NMTs and radiographers in preclinical imaging remains rare for various reasons:

- First, preclinical researchers are not aware of the existence of these professions and their level of expertise. Additionally, NMTs and radiographers may not be aware of the existence of such laboratories that could be potentially interested in their know-how.
- Second, integrating this kind of department requires the NMT or radiographer to leave their traditional work environment and consequently their comfort zone to take on new work habits and to assimilate new “codes” and new knowledge specific to the research sector.
- Third, the integration of an NMT or radiographer in a research unit is generally done on research funds and limited in time, depending on the projects of the laboratory. Consequently, the employment contracts offered are generally precarious because succession and accumulation of fixed-term contracts and subsequently obtaining a permanent professional situation remain difficult.
- Fourth, it may be challenging to obtain the same level of compensation as in the clinic.

## European Qualification Framework

In light of the current European Qualification Framework, a set of skills, knowledge and competencies were defined by the workgroup (Table 2). This description is considered to cover the whole set of duties and tasks deliverable to an NMT or radiographer working in a preclinical laboratory and should be attained with proper education and training (Fragoso Costa et al. 2017).

**Table 2:** Additional set of knowledge, skills and competencies for NMTs and radiographers working in preclinical imaging laboratory

**Knowledge**	**Skills**	**Competencies**
**K1**. Understand the importance of animal imaging in the pathway of drug discovery	**S1**. Collaborate in a multidisciplinary team in the implementation of animal scans in the context of drug development and research	**C1.** Take responsibility for the implementation and optimisation of animal imaging
**K2.** Know the legal and ethical ordinances that apply to the process of animal experimentation	**K2.** Follow the legal and ethical framework that are necessary for animal project licenses	**C2.** Critically review all processes of animal experimentation, taking into account the actual scientific, legal and ethical standards
**K3.** Possess knowledge on the biology and physiology of animal models	**S3.** Perform different radiopharmaceutical, pharmaceutical and contrast media administration routes on animal models	**C3.** Optimise imaging procedures taking into account the specific features of the animal model and administration route
**K4.** Understand the biological mechanisms underlying the process of animal anaesthesia	**S4.** Perform animal anaesthesia following the current evidence based guidelines	**C4.** Take accountability for the use of anaesthesia and report all such acts accordingly
**K5.** Know the underlying genetic principles on the breeding process	**S5**. Respect the current guidelines on the handling of both native and genetically modified animals	**C5**. Keep updated records on the handling and breeding of animal models
**K6.** Have knowledge on hygiene and microbiologic standardisation of experimental animal models	**S6.** Follow the current guidelines and local rules that apply to hygiene behaviour	**C6**. Take responsibility and keep records on the hygiene status of the laboratory
**K7.** General influence of environment factors on animal models and the subsequent imaging pattern	**S7.** Monitor the animal models before, during and after the imaging procedure	**C7.** Take responsibility and keep records on the possible factors that might affect imaging and the animal well-being
**K8.** Know the factors that affect animal welfare	**S8.** Follow and comply with the national law and international guidelines that apply to animal care and welfare	**C8.** Take full legal and professional responsibility for the condition of animal care and welfare on your laboratory
**K9.** Understand the principles of preclinical experimental design (3Rs) and reporting	**S9.** Comply with the most up-to-date guidelines and use the available tools for good experimental design and reporting	**S9.** Take accountability for transparency and good animal reporting

## Multimodal preclinical imaging lab: an opportunity for NMT and radiographer’s professional development and research?

A preclinical imaging laboratory can be widely considered as an opportunity for NMTs and radiographers to express their skills and develop specific research axes, particularly focused on technical aspects. This environment allows to initiate their own research projects concerning:

- Methodological developments
- Evaluations (new procedures, pharmaceutical agents or devices)
- Workflow organisation

There are many examples of studies carried out by NMTs and radiographers. For instance, one revealed specific absorption of a number of radiotracers in the ventricular system in rats (Zeilinger et al. 2017). Another one focuses on gadolinated agents’ injection pathways in preclinical MRI, taking into consideration the animal welfare and showing that subcutaneous routes could be an alternative to intravenous routes (Dillenseger et al. 2019).

But at this time, it is difficult to estimate the number of publications corresponding to studies carried out by NMTs and radiographers in the field of multimodal preclinical imaging, for several reasons:

- There are no journals dedicated to the NMT and radiographer community in the field of multimodal imaging.
- Strict evaluation of pragmatic methodological approaches does not necessarily fall within the scope of journals which are more focused on pre-clinical research results or on engineering development.
- When a study is published by a radiographer or NMT, its professional affiliation is not necessarily mentioned, and the degree (bachelor, master, PhD) will be given greater prominence.
- ilia. It is not easy to cover publication fees for NMTs and radiographers who are not engaged in an academic curriculum (e.g. PhD Fellow). Thus, a significant number of works are limited to congress communications and do not take the form of publications in peer-reviewed journals.

However, in a preclinical environment, NMTs and radiographers are the right people to occupy several interesting roles depending on their experiences and investments in the field:

- Carrying out procedures (anaesthesia, acquisition, processing, recovering)
- Organizational role (workflow management, quality control and maintenance programming)
- Research project facilitator (expert/researcher support)
- Research investigator in their own disciplinary fields

These roles highlight and valorise the skills of NMTs and radiographers. It is then possible to affirm that the field of preclinical imaging is promising for the radiographer and NMT professions, both in terms of professional development and applied research. There are no other professional curricula that are so close to the requirements of a preclinical imaging platform.

## Conclusion

The preclinical imaging field represents an interesting opportunity for medical technical professionals wishing to join a research team. The debates held at a European level (Dillenseger et al. 2014; Dillenseger 2018) demonstrate that the integration of NMTs and radiographers in preclinical labs works and allows for efficient use of resources, increasing the number and especially the quality of experiments performed. These successful integrations show that a set of knowledge, skills and competencies can be perfectly transferred from the clinic into preclinical imaging to improve and evaluate experimental procedures. Some evidence has also shown that an experienced NMT or radiographer in this sector can take on roles as research investigators (Zeilinger et al. 2017; Dillenseger et al. 2019). Involvement of NMTs and radiographers in new preclinical molecular and quantitative imaging methods, which are vastly growing fields, could be highly beneficial for their own professional development as a win-win complementary bridge with the clinical field (Dillenseger et al. 2019), but also could be an enabler for more fundamental works. We believe and have demonstrated that NMTs and radiographers’ skills match nearly perfectly with the requirements of a preclinical imaging lab, and that they could be considered a keystone of such organization in the future.
